# Surface-Shaving of *Staphylococcus aureus* Strains and Quantitative Proteomic Analysis Reveal Differences in Protein Abundance of the Surfaceome

**DOI:** 10.3390/microorganisms12081725

**Published:** 2024-08-21

**Authors:** Anders Karlsson, Leonarda Achá Alarcón, Beatriz Piñeiro-Iglesias, Gunnar Jacobsson, Susann Skovbjerg, Edward R. B. Moore, Pradeep Kumar Kopparapu, Tao Jin, Roger Karlsson

**Affiliations:** 1 Nanoxis Consulting AB, 40016 Gothenburg, Sweden; anders.karlsson@nanoxisconsulting.com; 2 Department of Infectious Diseases, Institute of Biomedicine, Sahlgrenska Academy, University of Gothenburg, 40530 Gothenburg, Sweden; leonarda.acha.alarcon@gu.se (L.A.A.); beatriz.pineiro.iglesias@vgregion.se (B.P.-I.); susann.skovbjerg@vgregion.se (S.S.); erbmoore@ccug.se (E.R.B.M.); 3 Department of Clinical Microbiology, Sahlgrenska University Hospital, Region Västra Götaland, 41345 Gothenburg, Sweden; 4 Department of Infectious Diseases, Skaraborg Hospital, 54185 Skövde, Sweden; gunnar.jacobsson@vgregion.se; 5 Centre for Antibiotic Resistance Research (CARe), University of Gothenburg, 40530 Gothenburg, Sweden; 6 Culture Collection of the University of Gothenburg (CCUG), Sahlgrenska Academy, 41390 Gothenburg, Sweden; 7 Department of Rheumatology and Inflammation Research, Institute of Medicine, Sahlgrenska Academy, University of Gothenburg, 41390 Gothenburg, Sweden; pradeep.kopparapu@gu.se (P.K.K.); tao.jin@rheuma.gu.se (T.J.); 8 Department of Rheumatology, Sahlgrenska University Hospital, 41345 Gothenburg, Sweden

**Keywords:** *Staphylococcus aureus*, proteomics, surfaceome, tandem mass tags, relative quantification, mass spectrometry, virulence factor

## Abstract

*Staphylococcus aureus* is a pathogen known to cause a wide range of infections. To find new targets for identification and to understand host–pathogen interactions, many studies have focused on surface proteins. We performed bacterial-cell surface-shaving, followed by tandem mass tag for quantitative mass spectrometry proteomics, to examine the surfaceome of *S. aureus*. Two steps were performed, the first step including surface protein-deficient mutants of *S. aureus* Newman strain lacking important virulence genes (*clfA* and *spa*, important for adhesion and immune evasion and *srtAsrtB*, linking surface-associated virulence factors to the surface) and the second step including isolates of different clinical origin. All strains were compared to the Newman strain. In Step 1, altogether, 7880 peptides were identified, corresponding to 1290 proteins. In Step 2, 4949 peptides were identified, corresponding to 919 proteins and for each strain, approximately 20 proteins showed differential expression compared to the Newman strain. The identified surface proteins were related to host-cell-adherence and immune-system-evasion, biofilm formation, and survival under harsh conditions. The results indicate that surface-shaving of intact *S. aureus* bacterial strains in combination with quantitative proteomics is a useful tool to distinguish differences in protein abundance of the surfaceome, including the expression of virulence factors.

## 1. Introduction

*Staphylococcus aureus* is a ubiquitous opportunistic human pathogen, carried asymptomatically by about 30% of the global population [1]. It is considered a versatile bacterium that can cause a broad array of infections, from shallow or mild skin infections (i.e., folliculitis, impetigo, etc.) to life-threatening conditions, such as bacteremia or sepsis. The emergence of methicillin-resistant *S. aureus* (MRSA) strains [2], its ability to obtain virulence-factor-encoding genes from the environment, as well as the scarcity of novel antibiotics, have spurred researchers to find alternative ways to eradicate infections caused by *S. aureus* [3,4] as well as to develop rapid and reliable diagnostics.

*S. aureus* is considered of high clinical relevance worldwide due to its role in nosocomial infections [5]; 40% of hospital-acquired diseases are caused by *S. aureus* [5]. Many infections might develop into bacteremia (*S. aureus* is the second most common cause of bloodstream infections) and sepsis [5,6,7]. Moreover, a delay in its detection and treatment might lead to mortality rates of 20% (sepsis), rising up to 80% in cases of severe sepsis and septic shock syndrome [7]; especially vulnerable are neonatal, elderly, and immunocompromised patients [5]. *S. aureus* strains are well known to manifest a wide variety of virulence factors [5,6] including surface proteins and toxins. Some surface proteins expressed by *S. aureus* play crucial roles in enabling the bacteria to adhere to the host cells, aid in invasion, and evade the immune response mounted by the host [7]. Surface proteins are known to mediate host–pathogen interactions, including adhesion or immune evasion, and have thus been main targets of vaccine development [8,9,10].

The *S. aureus* Newman strain was isolated from a human infection in 1952, displaying strong virulence traits and has been frequently used as a model strain due to its robust features [11,12]. Some surface proteins represent a key role in the adhesion to the host cells and immune evasion [8,13,14,15,16] and among these well-known proteins are Staphylococcal protein A (SpA) and Clumping factor A (ClfA) [17,18,19,20,21,22]. Mutants of the Newman strain created by deletion of *spa* and *clfa* genes are typically used to research virulence and immune evasion of *S. aureus* [18,20,23]. Some surface proteins of *S. aureus* are anchored to the bacterial cell wall by specific sortase enzymes. Two sortase enzymes, Sortase A (SrtA) and Sortase B (SrtB), play a main role during the staphylococcal infection [24,25,26]. Although mutants only lacking *srtB* gene still cause mild infections, mutants of *S. aureus* lacking both genes (Δ*srtAsrtB*) are known to display a reduced virulence and are commonly used to study skin infection and septic arthritis in in vivo models [25,27,28]. The surface proteins mentioned above are present in all *S. aureus* strains and some of them, such as SpA, are highly expressed [29,30].

Mass spectrometry (MS)-based proteomics has become a key tool for the study of biological systems. Recent developments in MS instrumentation, including Orbitrap technologies [31], have greatly improved its sensitivity, accuracy, and speed, and therefore the depth of the investigated proteomes [32]. Several thousands of proteins can be identified and quantified in a single analysis, enabling a comprehensive understanding of the expressed proteome of microorganisms [33,34].

Surface-shaving techniques followed by tandem MS analysis enable the study of the surfaceome [5,35,36,37,38,39,40,41,42,43,44]. As some of the previous references imply, a wide variety of methods are being used for surface-shaving; however, currently there is no “gold standard” methodology to target the bacterial surfaceome.

**Figure 1:**
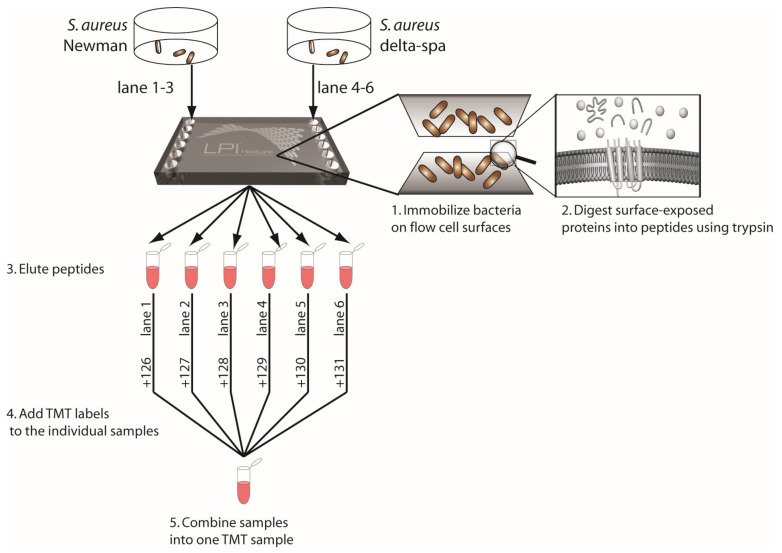
Schematic illustration showing the use of the Lipid-based Protein immobilization (LPI) methodology for performing surface-shaving of intact bacteria in combination with tandem mass tag (TMT) protocols.

Lipid-based Protein Immobilization (LPI^®^) methodology (Nanoxis Consulting AB, Gothenburg, Sweden) enabling surface-shaving of intact bacteria [42,45], in combination with relative quantification proteomics, using tandem mass tags (TMT) [46] (Figure 1), has previously shown valuable insight into the surfaceome [47,48]; however, to date, the combination of these techniques has never been used to study the relative abundance of surface proteins on *S. aureus* strains.

The use of knock-out strains as well as control strains commonly used in virulence studies provides a critical step forward in quantitative proteomic assessments. The knock-out strains verify correct proteomic analysis outcomes by demonstrating lack of target proteins. Well-characterized control strains with important virulence traits further provide an assessment of comparative expression of virulence factors especially significant when analyzing isolates of different clinical origin. This will provide a basis for improved understanding of the variation of expression of virulence traits for clinically relevant strains.

The first aim of this study was to use knock-out mutant strains of the Newman wildtype *S. aureus* strain to verify the absence of the gene deletions using the approach of combining LPI^®^ surface-shaving with comparative quantitative proteomics. The second aim was to investigate the relative expression of surface associated proteins in *S. aureus* clinical isolates of different origin and that display variances in pathogenicity.

## 2. Materials and Methods

### 2.1. Experimental Design

#### 2.1.1. Control Conditions: Selection of Buffer, Enzymes, and Digestion Time in a Pilot Step

The experimental design included a pilot step for the optimization of the surface-shaving technique, where some conditions such as buffers, enzyme concentration, and digestion time were assessed to determine the optimal conditions. Two buffers were tested: Phosphate buffered saline (PBS, pH 7.4) (Sigma-Aldrich, St. Louis, MO, USA) and 100 mM Triethylammonium bicarbonate (TEAB, pH 8.5) (Sigma-Aldrich, St. Louis, MO, USA) (a commonly used buffer for quantitative proteomics), where PBS is the mildest condition and TEAB the harshest.

#### 2.1.2. Control Procedure: Absence of the Proteins Linked to the Knock-Out Genes

Once the optimal surface-shaving was determined, two separate procedures (Step 1 and Step 2) were performed, which included the Newman strain as a particularly virulent strain, knock-out mutants thereof, as well as clinical strains (Table 1). The first step of the study aimed to confirm the absence of the proteins linked to the knock-out genes in the Newman mutant strains vs. the Newman strain.

In both steps, the surfaceome was analyzed using surface-shaving in combination with relative quantitative MS and tandem mass tags (TMT) (Figure 1). Finally, a qPCR was performed, to verify the proteomics results from Step 2, targeting the genes encoding for the most differentially expressed proteins (Clumping factor A and Staphylococcal Protein A).

#### 2.1.3. Control Strains: Step 1—The Protein Products of Knock-Out Mutant Strains Are Absent

*S. aureus* Newman strain was used as the reference strain and the “Mutant strains step” (Step 1), aimed to validate the surface-shaving methods using several well-characterized genetically modified *S. aureus* mutant strains lacking certain surface proteins. The study included the *S. aureus* Newman strain (also denoted CCUG 74308), from herein called “Newman” [11,12] and three surface protein-deficient mutant derivatives from the Newman strain; the Sortase A- and B-deficient mutant, Δ*srtAsrtB*, SKM14 (also denoted CCUG 74307) [25,27], herein called “ΔsrtAsrtB”; the Clumping factor A-deficient mutant, Δ*clfA*, DU5876 (also denoted CCUG 74305) [17,19,23], from herein called “ΔclfA”; and the Staphylococcal protein A-deficient mutant, Δ*spa*, DU5873 (also denoted CCUG 74306) [20,22], from herein called “Δspa” (Table 1).

#### 2.1.4. Step 2: Strains of Different Clinical Origin and Pathogenicity

The “Clinical strains step” (Step 2), included again the *S. aureus* Newman strain, two *S. aureus* well known reference strains, LS-1 (also denoted CCUG 74304) [21,49], from here on called “LS-1“ and SH1000 (also denoted CCUG 74303) [50,51], from here on called “SH1000”; and three *S. aureus* clinical strains isolated from patients, one strain causing mild skin and soft tissue infections, CCUG 74311 and two strains causing severe invasive diseases CCUG 74309 and CCUG 74310 (described in [52] (Table 1).

### 2.2. Cultivation of Bacteria and Preparation of Samples

The strains were grown on Columbia agar supplemented with 5% of defibrinated horse blood at 37 °C overnight. Bacterial biomass was collected in exponential phase growth (phase growth determined during a previous experimental design) and resuspended in phosphate-buffered saline (PBS). Bacterial suspension densities (Optical Density, OD) were measured using a spectrophotometer (WPA CO 8000 Cell Density Meter, Biochrom Ltd., Cambridge, UK) at a wavelength of 600 nm. For each experiment, the same amount of bacterial biomass was established by adjusting the OD to 1.0 in 1.0 mL of PBS, with corresponds approximately to 1 million bacteria (approximate CFU/mL was determined during a previous experimental design). Thereafter, the samples were washed three times with PBS, with centrifugation at 12,000× *g* during 5 min, discarding the supernatant and resuspending the pellet in 1.0 mL of PBS. The final bacterial pellets were resuspended in 150 µL of PBS. All samples were prepared in triplicate.

### 2.3. RNA Isolation and qPCR

The RNAs of all *S. aureus* strains were isolated by mechanical lysis with the Quick-RNA Fungal/Bacterial Miniprep kit (Zymo Research, Irvine, CA, USA) according to the manufacturer’s instructions, quantity and quality of RNA was measured with Nanodrop (ThermoFisher scientific, Waltham, MA, USA).

A quantitative PCR (qPCR) was performed as described by [53] (Supplemental Information File S1). Briefly, cDNA was synthesized with iScript™ cDNA Synthesis Kit (Bio-Rad, Hercules, CA, USA) as indicated by the manufacturer; the products were kept at −20 °C until qPCR reaction. For the quantification, two targets were selected, *spa* and *clfA*, the reactions were performed with TaqMan gene expression assays (Applied Biosystems, Warrington, UK), in a ViiA 7 Fast real-time PCR system (Applied Biosystems, Warrington, UK). GyrB was used as an internal control to normalize the data and the ΔΔCt method was used to obtain the relative abundances of the targets. The samples were analyzed in triplicates.

### 2.4. Lipid-Based Protein Immobilization and Digestion of Proteins into Peptides

A volume of 45 µL of each bacterial suspension was injected, with a pipette, into the LPI^®^ Hexalane FlowCell (Nanoxis Consulting AB, www.nanoxisconsulting.com (accessed on 20 August 2024)), filling the FlowCell channel. The intact bacterial cells were allowed to immobilize on the surfaces during 30 min at room temperature. Right after the incubation period, in order to remove the unbound bacteria, the FlowCell channels were washed with 100 µL of PBS, using a syringe pump, at a flowrate of 50 µL/min. Afterwards, enzymatic digestion of the bacterial surface proteins was performed by injecting 80 µL of trypsin solution (20 µg/mL in PBS) into the FlowCell channels, using the same syringe-pump setup, and incubating for 20 min at room temperature; then, excess trypsin solution was removed from the flow cell ports. The generated peptides were eluted by injecting 200 µL of PBS into the FlowCell channels and collected at the outlet ports, by means of a pipette. The samples were acidified using 4 µL of formic acid (neat). The peptide samples were subsequently centrifuged at 12,000× *g* during 5 min to remove any debris or detached bacteria, and the supernatants were stored at −20 °C until further MS analyses.

### 2.5. Liquid Chromatography and Tandem Mass Spectrometry (LC-MS/MS)

The proteomic analysis was performed at The Proteomics Core Facility at The Sahlgrenska Academy, University of Gothenburg. For the identification study (pilot step), digested peptides were desalted using Pierce C-18 spin columns (Thermo Scientific) according to manufacturer’s instruction. Digested peptides were labeled, using TMT 10-plex (Thermo Fisher Scientific), according to the manufacturer’s instructions (Step 1 and 2). The samples in Step 1 were divided into three separate TMT sets, with one of the triplicates in each set. The samples in Step 2 were divided into two TMT sets, whereby each set included a reference pool. The reference pool consisted of two additional preparations of the *S. aureus* Newman reference strain, pooled and divided into two identical samples. The TMT sets were fractionated into twelve fractions, using the Pierce High pH Reversed-Phase Peptide Fractionation Kit (Thermo Fisher Scientific), according to the manufacturer’s protocol but with a modified gradient (Supplemental Information File S2). The samples and fractions were dried, reconstituted in 3% acetonitrile, 0.1% formic acid and analysed on an Q Exactive (pilot experiment) or Orbitrap Fusion Tribrid mass spectrometer (Step 1 and 2) interfaced with nLC 1200 liquid chromatography system (Thermo Fisher Scientific). Peptides were trapped on an Acclaim Pepmap 100 C18 trap column (100 μm × 2 cm, particle size 5 μm; Thermo Fischer Scientific) and separated in an in-house-constructed analytical column (300 × 0.075 mm I.D.) packed with 3 μm Reprosil-Pur C18-AQ particles (Dr. Maisch, Ammerbuch, Germany), using a linear gradient of 7% to 80% acetonitrile in 0.2% formic acid over 90 min. On the Q Exactive precursor ion mass spectra were recorded at 70,000 resolution. The 10 most intense precursor ions were fragmented using HCD at collision energy setting of 30 spectra and the MS/MS spectra were recorded at 35,000 resolution. Charge states 2 to 6 were selected for fragmentation, dynamic exclusion was set to 20 s. In TMT studies precursor ion mass spectra were acquired at 120,000 resolution and MS/MS analysis was performed in a data-dependent multinotch mode. Charge states 2 to 7 were selected for fragmentation, with an isolation window of 0.7 *m*/*z*, and dynamic exclusion was set to 60 s and 10 ppm. MS3 spectra for reporter ion quantitation were recorded at 60,000 resolution with HCD fragmentation at collision energy of 55 using the synchronous precursor selection.

### 2.6. Protein Identification and Quantification

The data files for each set were merged for identification and relative quantification, using the Proteome Discoverer 1.4 software (Thermo Fisher Scientific). The search was matched against *S. aureus* Newman genomes (downloaded from NCBI on 11 December 2017, 2856 protein entries, Supplemental Information File S3) using Mascot 2.5 (Matrix Science) as a search engine, with precursor mass tolerance of 5 ppm and fragment mass tolerance of 200 mmu (pilot step) or 0.6 Da (Step 1 and 2). Tryptic peptides were accepted with one missed cleavage and variable modifications of methionine oxidation and additional fixed TMT-label modifications of N-terminal and lysine for TMT studies were selected. In Step 1, the Newman samples in each TMT set were used as the denominator and for calculation of the ratios, while in Step 2, the reference samples were defined as the denominator. Target Decoy (pilot experiment) were used for the PSM validation, peptides were filtered at 1% FDR (False Discovery Rate) and grouped by sharing the same sequences to minimize redundancy. No normalization was applied in the TMT studies, fixed Value for PSM Validation, replace-missing values and to increase the quality of data Minimum Quan Value Threshold was set to 1500. Only peptides unique for a given protein were used for quantification. Protein quantification ratios were calculated from peptides quantification ratios by taking the median values to reduce the impact of outliers.

### 2.7. Functional Annotation

The proteome of *S. aureus* Newman strain was analyzed using OmicsBox v3.2.9 (BioBam Bioinformatics S.L., Valencia, Spain). Briefly, proteins were classified into Gene Ontology (GO) terms by eggNOG-Mapper v2.1.0, IterProScan v5.68-100.0 and Blast2GO GOA version 2022.08. Only terms based on one-to-one ontology and experimental evidence were considered in eggNOG-Mapper annotation. Annotation results were filtered to remove redundancy using the GO True Path Rule and the Class Bacilli (Taxomony ID: 91061). The filtered results were assigned to Enzyme Commission (EC) numbers, and metabolic pathways were annotated with eggNOG v5.0.2 using these EC numbers and the Kyoto Encyclopedia of Genes and Genomes (KEGG) (ref) in OmicsBox v3.2.9.

### 2.8. Statistical Evaluation

The raw data from the MS analyses were log2-transformed, quantile-normalized, and batch-corrected. The normalize.quantiles function in the R-package preprocessCore and the ComBat function in the R-package sva were used. Welch’s *t*-test was used to test for differences in abundance (log2-transformed) between the groups. The data was also evaluated by a gene significance score called π-value [54]), taking into consideration both the fold change and the *p*-value. This approach allows proteins with large fold changes, but non-significant *p*-values, however passing the π-value, to be included in further analyses, since they might be of biological relevance.

**Figure 2:**
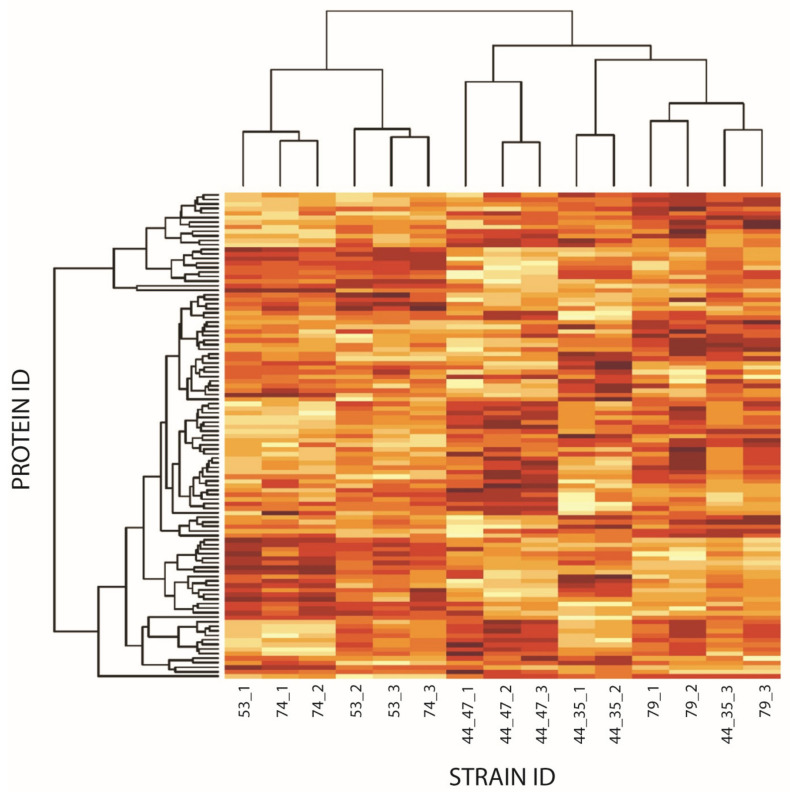
Heatmap showing the protein abundance of two clinical reference strains LS-1 (53) (1,2,3) and SH1000 (74) (1,2,3), and three clinical strains CCUG 74309 (44_35) (1,2,3), CCUG 74310 (44_47) (1,2,3) and CCUG 74311 (79) (1,2,3) when compared to the Newman strain. The *y*-axis shows the protein IDs, whereas the *x*-axis shows the strain ID number for each individual strain analysis performed in triplicates. Red colour indicates a higher abundance, whereas yellow signifies a lower abundance (fold change (FC) of strain compared to Newman). Clustering regarding similarity in high or low abundance as compared to the Newman strain was allowed for the protein identification (PROTEIN ID) but was also allowed for the samples (STRAIN ID), strains showing similar patterns in the protein abundance profiles cluster together. Here, in this cluster analysis, only values passing *p*-values < 0.05 from the ANOVA analysis is included.

The heatmaps (Figure 2, Supplemental Information Files S4 and S5) are made in R and the clustering algorithm that the heatmap function uses is called hierarchical clustering. The hierarchical clustering clusters both samples and proteins based on their expression profiles, so proteins with similar profiles fall close to each other and samples with similar profiles fall close to each other. The colors, yellow and red, show row-normalized expression values where yellow represents low expression and red high expression.

## 3. Results

### 3.1. Pilot Experimient—Surface-Shaving Optimization: PBS Is the Most Suitable Buffer to Perform Surface-Shaving of Intact S. aureus

A qualitative pilot experiment was used to optimize the method before performing quantitative analyses. General protein identification was performed, testing various conditions, in two selected strains (Newman and Δ*spa* strain).

Table 2 shows selected results from varying buffer composition (from PBS to TEAB), while, the full panel of results can be found in Supplemental Information File S6. The differential number of proteins (identified with more than two peptides) detected in both strains, shifts from tens of proteins in presence of 100% PBS (10 for Newman and 13 for Δ*spa*), up to nearly hundred proteins with 100% TEAB (89 and 96, respectively). The increase in the number of detected proteins with harsher buffer conditions (TEAB), is most likely due to an increase in the number of cytosolic proteins signifying a higher degree of lysis during digestion (Table 2).

The proportion of cytosolic proteins obtained with TEAB was similar to the number of cytosolic proteins observed in the whole cell digest. Meanwhile, the proteins identified by surface-shaving had a smaller number of cytosolic proteins (Table 2).

Most of the identified proteins were surface associated proteins (according to annotation through PSORTb [55], but also the so-called moonlighting proteins [56], such as the Elongation factor Tu (EF-Tu), known to also play a role on the surface, acting as a virulence factor [57].

### 3.2. Step 1-Mutant Strains Step: The Protein Products of the Knocked-Out Genes Were Not Identified in the Mutant Strains by Surface-Shaving

Based on the results from the pilot experiment, PBS was chosen as the digestion buffer to reduce the degree of lysis during the digestion and the presence of cytosolic proteins.

Step 1 included three separate TMT-sets. In set 1 (Δ*clfA* vs. Newman), 5532 peptides were identified matching against the *S. aureus* Newman genome data bases, corresponding to 1104 proteins. In set 2 (Δ*srtAsrtB* vs. Newman) and set 3 (Δ*spa* vs. Newman), 6354 and 5596 peptides were identified, corresponding to 1176 and 1088 proteins, respectively. Altogether, 7880 peptides were identified in this procedure, corresponding to 1290 proteins (Supplemental Information File S7). The differences in protein abundance were visualized in a heat map (Supplemental Information File S4). All the strains were analyzed in triplicates and compared against the Newman strain. The heat map was generated by allowing samples (strain vs. Newman) to cluster according to how similar were to each other, i.e., not having a fixed sample order. The identified proteins were further annotated by OmicsBox. The results shown in Table 3 are only the ones passing the threshold of FC of +/−2 and *p*-values < 0.05, showing the differential abundance in the proteins (FC) of the mutants when compared to the Newman strain. The Δ*clfA* mutant was lacking the ClfA protein (FC −24) when compared to the Newman strain, as expected; without relative changes in abundance of SpA and other cell wall proteins like Clumping factor B (ClfB) and plasma membrane protein MAP-ND2C (Table 3); whereas in set 2 (Δ*srtAsrtB* mutant vs. Newman), the SpA, both the Clumping factors A and B and the MAP displayed lower relative abundance than the Newman parental strain (FCs −11, −10, −3, −8, respectively). Furthermore, a capsid protein and some hypothetical proteins of unknown function displayed similar pattern of lower expression. The absence of these proteins can be explained as a mutant strain lacking both sortases (Δ*srtAsrtB*) is deficient in the assembly of several surface proteins, such as Staphylococcal Protein A (spa) and Clumping factors A and B (ClfA and ClfB), failing in the anchoring of adhesins to the cell wall [25,27]. Lastly, as expected, the Δspa mutant (set 3) demonstrated the absence of the SpA (FC −25). Notwithstanding, a small number of the expressed proteins that were less abundant in the Δ*srtAsrtB* mutant, showed an opposite pattern, increasing their relative abundance in the Δ*spa* mutant when compared with WT, i.e., the well-known protein ClfB with FC 2 in Δ*spa* and FC −3 in Δ*srtAsrtB* (Table 3). This also occurred with two hypothetical proteins, further annotated by OmicsBox (accession numbers WP_000438352.1 (phage protein) and WP_001175781.1 (aminotransferase class IV)), with FC 9 and FC 2, respectively in Δ*spa*, but a decline in the sortase mutant with FC −3 and −2, respectively. The relative abundance of previously mentioned proteins does not pass the threshold in the Δ*clfA* mutant; therefore, the relative abundance was not statistically relevant (Supplemental Information File S7).

Results from Step 1 (Mutant strains step), verifying knock-out of Clumping factor A (Δ*clfA*) and Staphylococcal protein A/Immunoglobulin G-binding protein A (Δ*spa*), shown by the absence of these proteins in the TMT analysis. Also shown is the knock-out of sortase (Δ*srtAsrtB*), resulting in lower abundance of several surface-associated proteins, some of which were demonstrating higher abundance in the Δ*spa* mutant (e.g., ClfB). Proteins in the list were sorted by the fold change of the Δ*srtAsrtB* mutant, showing the proteins that had lower abundance level when compared to the Newman strain. Only fold changes (FC) of +/−2 passing *p*-values < 0.05 were included. “−” indicates no change in abundance when compared to the Newman strain.

### 3.3. Step 2—Clinical Strains Step: Differential Protein Expression Is Observed in the Clinical Strains as Compared to the Virulent Newman Strain

A total of 4949 peptides were identified in Step 2 corresponding to 919 proteins. The differences in protein abundance were visualized in a heat map (Figure 2). All the strains in this study were analyzed in triplicates and compared against the Newman strain (Figure 2, Supplemental Information File S8). The heat map was generated by allowing samples (strain vs. Newman) to cluster according to how similar were to each other, i.e., not having a fixed sample order. Both reference strains showed similar trends, and therefore cluster into one group; while the three clinical strains cluster together into another group, distinct from the reference strains, as all clinical samples follow a similar pattern when compared to Newman (Figure 2, Table 4, Supplemental Information File S5). After analyzing the results (Supplemental Information File S8, Table 4); several proteins follow a similar trend in all the strains, i.e., ClfA, in both reference strains (LS-1 and SH1000), and clinical strains vs. Newman, the relative abundance of ClfA was decreased (FCs −4 and −3 for the reference strains) and even more pronounced reduction in clinical strains (FC −10, −8, −16). Similar trends were observed for proteins Efb-c, MAP-N2C protein, and SdrE (Table 4). Other proteins, however, exhibited an increase in all strains e.g., EbpS (FC 2) and an uncharacterized protein from the Asp23/Gls24 family (FC 2, 3). Some proteins displayed different expression pattern when comparing the reference strains vs. Newman and clinical isolates vs. Newman. One of these proteins was SpA, which was less expressed in LS-1 and SH1000 compared to Newman (FCs −4, −2, respectively), however, in the clinical strains a higher relative abundance in the expression was observed (FCs 2, 2, and 3, respectively). Lastly, Emp and Sbi proteins are slightly increased in the reference strains (FC 2) and slightly reduced in the clinical isolates (FC −2) (Table 4). The identified proteins were further annotated using OmicsBox (Supplemental Information File S8).

### 3.4. Comparison of Both Steps: Mutant Strains and Clinical Isolates Vs. Newman Strain

In Step 1, several proteins exhibited higher relative expression in the mutants when compared to the Newman strain, and only few proteins displayed lower relative expression when compared to the Newman strain (Table 3 and Supplemental Information File S7).

**Figure 3:**
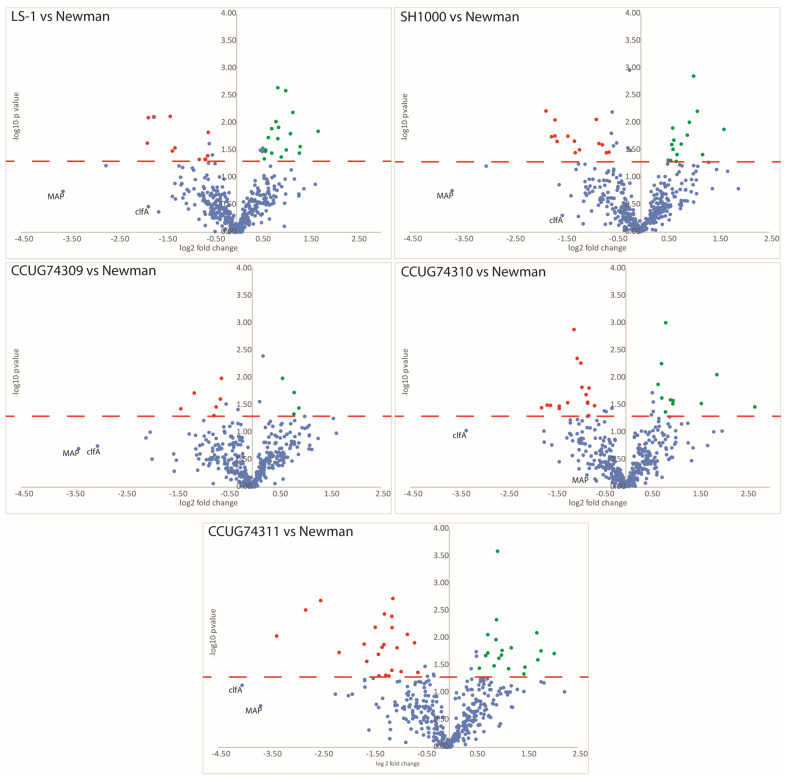
Volcano plots (−log10 *p*-values vs. log2 fold changes) for the reference strains LS-1, SH1000, as well as three clinical strains (CCUG74309 (invasive), CCUG74310 (invasive) and CCUG74311 (skin infection)) all vs. Newman *S. aureus* strain. Red dotted line signifies the cut-off for *p*-value < 0.05, values above dotted line are statistically significant. Of the proteins demonstrating statistically significant fold changes, the dots in red signifies proteins with fold changes <−1.5 (downregulated) whereas dots in green signifies proteins with fold changes >1.5 (upregulated), blue dots signify proteins not passing the cut-offs of fold change or p-value. The Clumping factor A (cflA) and MAP-domain-containing protein are marked in each sub-panel showing the similar trends of lower expression (these pass the significance threshold when using the π-value as described in the text).

In Step 2, the differences were more evenly distributed regarding differential relative expression of the proteins. For each strain, approximately 20 proteins showed higher or lower levels of expression when compared to the Newman strain. Clinical strain, CCUG 74311, from mild skin infection, displayed the highest number of proteins being differentially expressed when compared to Newman (Table 4 and Supplemental Information File S8). Volcano plots (plotting the statistical significance (*p*-values) vs. fold change (log2)) were generated for all the pair-wise comparisons vs. the Newman strain for Step 2 (Figure 3). For Step 2, the important virulence factors (Clumping factor A and the MAP-domain containing protein) stand out by displaying much lower abundance when compared to the Newman (fold changes ranging from −2 to −16). Statistically, these two proteins do not pass the *p*-value restriction (*p* < 0.05); however, as the trends clearly show a strong decrease in abundance, the data were also processed by implementing the π-value [54], considering that large fold changes might still have biological importance despite large *p*-values (Supplemental Information Files S7 and S8).

Two clinical reference strains (LS-1 and SH1000) as well as three clinical strains (CCUG 74310, CCUG 74309 and CCUG 74311) compared to the Newman strain, showing fold changes (FCs) of proteins, demonstrating differential protein abundance. A selection of proteins displaying the highest fold changes (higher or lower abundance), sorted according to the fold changes observed for the comparison between strain CCUG 74311 and the Newman strain, are shown. Only fold changes passing pi-values of 1 were included.

### 3.5. Clinical Step Results and qPCR—The Expression Trends of Genes and Proteins Are in Accordance within the Clinical Strains

**Figure 4:**
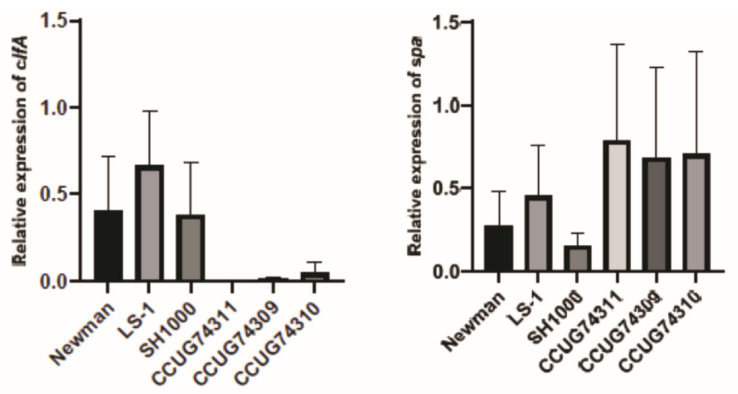
Relative abundance in the expression of targeted genes, *clfA* and *spa,* in Newman strain vs. reference strains (LS-1 and SH1000), and clinical strains of diverse origin (CCUG 74309, CCUG 74310 and CCUG 74311) obtained by qPCR methodology. *Y*-axis represents relative abundance of the selected protein, while *x*-axis represent the bacterial strains.

Two of the differentially expressed proteins (SpA and ClfA) found in Step 2 were selected as targets to perform qPCR and compare their relative gene expression to their protein expression detected by surface-shaving and quantitative proteomics. Compared to the Newman strain, the gene expression of *clfA* in all the clinical strains and SH-1000 was downregulated (Figure 4), which is consistent with the proteomic surface-shaving results (Table 4) where ClfA demonstrated lower expression. The gene expression of *clfA* in LS-1 on the other hand contrasted the results obtained by proteomics. Although the gene and protein expressions followed the same trends for most of the strains tested no statistical significance (*p* < 0.05) was found (Table 4 and Figure 4).

Regarding the gene expression of *spa* in the clinical strains, an upregulation of the target was observed compared to the Newman strain, which could be verified with the proteomic results (Table 4 and Figure 4). For the strain SH-1000, a slight downregulation in gene expression was recorded, keeping the trend observed in the results of Step 2. However, the LS-1 strain showed non-consistent results between the two methods, upregulation of the *spa* gene was observed when compared to the Newman strain in the qPCR, while the proteomic surface-shaving results indicated downregulation of the protein. No statistical significance was, however, observed.

## 4. Discussion

The purpose of this study was not to validate the LPI methodology combined with surface-shaving, nor to appoint this combined methodology as a “gold standard”, but rather to demonstrate the feasibility of the approach for studying the surfaceome of *S. aureus*. An important advantage with the LPI HexaLane FlowCell, is that very short digestion times can be implemented, enabling digestion of the surface exposed proteins, thus highlighting the surfaceome.

### 4.1. Mutant Strains: Plausible Cascade Pattern with Compensational Mechanisms after Deleting Crucial Genes

In all three knock-out mutants, several proteins displayed higher expression, (FC > 2 vs. Newman) (Supplemental Information File S7) for instance, in the Δ*clfA* mutant (lacking ClfA protein), iron-regulated surface proteins (IsdA and IsdB) and penicillin-binding proteins demonstrated higher relative abundance than in the Newman strain (Supplemental Information File S7). In contrast, the Δ*spa* mutant (lacking SpA) displayed higher abundance of Clumping factor B along with Elastin-binding proteins and Iron-regulated surface protein IsdA. For the Δ*srtAsrtB* mutant (lacking both sortase proteins), key proteins involved in virulence, such as SasD, Esa, SdrE, Sbi and penicillin-binding proteins (PBP) displayed higher expression and Clumping factor B lower expression. The complex pattern of diverging expression was visualized in a heat map (Supplemental Information File S4). According to the results, it could be interpreted that possible compensational mechanisms took place in the *S. aureus* mutant strains, when deleting targeted genes; highlighting that certain surface proteins were potentially up/downregulated, after a complex cascade of events, due to the deletion. Therefore, it is plausible, that an intricate pattern of surface proteins rather than one single virulence factor (surface protein) should be considered in data interpretation as well as in vivo studies (where surface protein-deficient mutants are commonly used); and a panel of virulence factors, rather than one single protein, might be determinants for the virulence of certain type of infections.

### 4.2. Clinical Strains: The Clinical Strains Displayed Higher Protein Expression of SpA, a Key Virulence Factor Involved in S. aureus Immune Evasion

Changes in differential expression of certain surface proteins were observed when comparing reference strains and clinical strains vs. Newman. Staphylococcal protein A (SpA), a highly conserved multifunctional key virulence factor, said to be involved in the immune evasion of *S. aureus*, due to its ability to bind the Fc portions of IgG antibodies [58], displayed higher expression in the three clinical isolates when compared to Newman. In contrast, the reference strains displayed a lower expression of Staphylococcal protein A (SpA) as compared to Newman (Table 4). On the other hand, the Extracellular matrix protein-binding adhesin (Emp) and the Immunoglobulin-binding protein (Sbi), showed a decrease in their expression in the clinical strains vs. Newman while an increase in the reference strains vs. Newman. Emp is a secreted adhesin that binds to the host cell proteins fibronectin, fibrinogen, collagen and vitronectin [59] and Sbi has been shown to be involved in immune evasion [11,60] and play a role in later stages of the infection [59].

However, there were also some common trends between reference and clinical strains, for example, the MAP domain-containing protein as well as the Clumping factor A protein both showed much lower abundance in all five strains, indicating that these two proteins are very much abundant in the Newman strain, when compared to the strains included in this analysis (Table 4). Both the MAP domain-containing protein and the multifunctional Clumping factor A are important virulence factors involved in immunomodulation [8,61] as well as adhesion [61,62] and are mainly expressed during growth phase.

Different culture conditions and/or growth phases have not been investigated at this stage, neither proteins secreted to the extracellular space, since secreted proteins that are not attached to the cell surface will not be detected by this methodology, neither different host environments. A broader approach including different condition set-ups will be pursued in follow-up studies applying the methodology as described in [45].

Surface-associated proteins, such as the autolysin, the Staphylococcal protein A, clumping factors A and B, iron-regulated immuno-dominant antigens A and B, among others, were identified with several peptides (and PSMs) in the qualitative pilot experiment (Table 2) and in Step 1 and 2 (Table 3 and Table 4). However, proteins not associated with the surface were also identified. This outcome is not surprising, since the approach used for the quantitative TMT-based proteomics in Step 1 and 2, that is, multiplexing of samples in combination with pre-fractionation, will significantly improve the number of identified and quantified proteins. Pre-fractionation reduces the sample complexity and therefore less peptides are co-eluting, which facilitates the detection of lower abundant peptides. In addition to reduced sample complexity, the amount of starting material is increased, for example 10 times when analyzing a TMT-10plex, as compared to analyzing the samples individually. Thus, the number of low abundant proteins that are not associated with the surface will be increased.

## 5. Conclusions and Next Steps

Our data suggest that the strategy of using surface-shaving (LPI HexaLane FlowCell), combined with relative protein quantification (TMT), could be a useful tool to study the relative abundance of surface proteins in S. *aureus* strains.

Further studies, including a higher number of clinical strains, should be performed to obtain statistically significant results and evaluate which combination of virulence factors could be potentially used as future targets for treatments [2] and/or rapid identification methods.

## Figures and Tables

**Table 1:** Description of the *Staphylococcus aureus* strains included in Step 1 and 2.

Step 1—Mutant Strains		Step 2—Clinical Strains	
Strain	Description	Strain	Description
CCUG 74308 “Newman”	Newman strain (Foster lab), Duthie and Lorenz 1952; Baba et al., 2008 [11,12]	CCUG 74308 “Newman”	Newman strain (Foster lab), Duthie and Lorenz 1952; Baba et al., 2008 [11,12]
CCUG 74306 “∆s*pa*”	Newman Δ*spa* DU5873 Newman strain (Foster lab) knock-out mutant of Staphylococcal protein A, Kim et al., 2011; Kobayashi and DeLeo 2013 [20,22]	CCUG 74304 “LS-1”	LS-1 Bremell et al., 1994; Verdrengh and Tarkowski 1997 [21,49]
CCUG 74305 “∆*clfA*”	Newman Δ*clfA* DU5876 Newman strain (Foster lab) knock-out mutant of Clumping factor A, McDewitt et al., 1994, 1995; Higgins et al., 2006 [17,19,23]	CCUG 74303 “SH1000”	SH1000 Horsburgh et al., 2002; O’Neill 2010 [50,51]
CCUG 74307 “Δ*srtAsrtB*”	Newman Δ*srtAsrtB* SKM14 Newman strain Schneewind lab knock-out mutant of Sortase A and B, Mazmanian et al., 2000; Jonsson et al., 2003 [25,27]	CCUG 74309	Invasive strain, 44_35 Jacobsson et al., 2007 [52]
		CCUG 74310	Invasive strain, 44_47 Jacobsson et al., 2007 [52]
		CCUG 74311	Clinical strain from mild skin and soft tissue infection, 79 Kwiecinski et al., 2014 [16]

**Table 2:** Qualitative proteomics results from surface-shaving of intact cells at different buffer conditions.

Accession NCBI	Protein Names from *S. aureus* Newman (NCBI Name)	Gene Names from *S. aureus* Newman	Subcellular Location (PSORTb)	NM PBS	Δ*spa* PBS	NM 10% TEAB	Δ*spa* 10% TEAB	NM 25% TEAB	Δ*spa* 25% TEAB	NM 100% TEAB	Δ*spa* 100% TEAB
WP_000728763.1	Staphylococcal protein A (Spa)	*spa* NWMN_0055	Cellwall	8	0	12	0	11	0	14	0
WP_001074508.1	Bifunctional autolysin	*atl* NWMN_0922	Extracellular	7	12	18	12	13	11	27	25
WP_000215236.1	Asp23/Gls24 family envelope stress response protein	NWMN_2086	Unknown	5	7	6	4	7	7	7	6
WP_001056178.1	MSCRAMM family adhesin clumping factor A ClfA	*clfA* NWMN_0756	Cellwall	4	4	5	5	5	4	7	7
WP_001040568.1	Elongation factor Tu (EF-Tu)	*tuf* NWMN_0510	Cytoplasmic *	3	4	5	3	4	4	7	8
WP_001041586.1	Heme uptake protein IsdB	*isdB frpB sasJ sirH* NWMN_1040	Cellwall	3	5	6	3	4	3	11	10
WP_001549158.1	Extracellular adherence protein Eap/Map	NWMN_1872	Cytoplasmic membrane	3	2	9	3	6	4	13	10
WP_000383814.1	DUF948 domain-containing protein	NWMN_1632	Cytoplasmic	2	3	3	1	4	3	7	5
WP_000745871.1	MSCRAMM family adhesin clumping factor B ClfB	*clfB* NWMN_2529	Cellwall	2	2	3	1	3	2	5	3
WP_000160859.1	LPXTG-anchored heme-scavanging protein IsdA	*isdA frpA stbA* NWMN_1041	Cellwall	2	3	3	3	2	3	4	3
			Σ Proteins > 2 peptides	10	13	26	11	28	23	89	96

Qualitative proteomic results of Nemwan (NM) and knock-out spa mutant (Δ*spa*). The number of peptides identified per condition is shown. The proteins were ranked by the number of peptides found for the mildest surface-shaving condition (PBS), only proteins identified with >2 peptides for this particular condition are shown. The surface-shaving performed at PBS only is the mildest condition for the cells, whereas an increase in TEAB going from 10%, 25% up to 100% is a harsher condition. For Steps 1 and 2, the mildest condition, digestion using PBS only, was used. Subcellular location predicted by PSORTb (Yu et al., 2010 [55]) is shown. PBS stands for phosphate-buffered saline while TEAB is triethylamonium bicarbonate buffer. * Although predicted by PSORTb to have a Cytoplasmic subcellular location, EF-Tu has been shown to also be present on the surface of the bacterial cells (extracellular), as a “moonlighting” protein, playing a role in the pathogenesis of the bacteria.

**Table 3:** Differential expression of knock-out proteins in the mutant strains vs. Newman (Step 1).

Accession Number	NCBI Protein Name	Protein Names from *S. aureus* Newman	Subcellular Location	Gene Name from *S. aureus* Newman	Fold Change (FC) vs. Newman		
					∆*clfA*	Δ*srtAsrtB*	∆*spa*
WP_000728763.1	Staphylococcal protein A	Immunoglobulin G-binding protein A (IgG-binding protein A) (Staphylococcal protein A) (Spa)	extracellular region; cell wall; membrane	*spa* NWMN_0055	-	−11	−25
WP_001056178.1	MSCRAMM family adhesin clumping factor ClfA	Clumping factor A (Fibrinogen receptor A) (Fibrinogen-binding protein A)	extracellular region; cell wall; membrane	*clfA* NWMN_0756	−24	−10	-
WP_001549158.1	Extracellular adherence protein Eap/Map	65 kDa membrane protein (map-ND2C)	plasma membrane	NWMN_1872	-	−8	-
WP_000745871.1	MSCRAMM family adhesin clumping factor ClfB	Clumping factor B (Fibrinogen receptor B) (Fibrinogen-binding protein B)	extracellular region; cell wall; integral component of membrane	*clfB* NWMN_2529	-	−3	2

**Table 4:** Differential protein expression in reference strains and clinical strains vs. Newman strain.

Accession Number	NCBI Protein Name	Protein Name from *S. aureus* Newman	Subcellular Location	Gene Name from *S. aureus* Newman WT	Fold Change (FC) vs. Newman				
					LS-1	SH1000	CCUG 74310	CCUG 74309	CCUG 74311
WP_000728763.1	Staphylococcal protein A	Immunoglobulin G-binding protein A (IgG-binding protein A) (Staphylococcal protein A) (Spa)	extracellular region; cell wall; membrane	*spa* NWMN_0055	−4	−2	2	2	3
WP_000241588.1	Asp23/Gls24 family envelope stress response protein	Uncharacterized protein		NWMN_1430	2	2	3	2	3
WP_000069282.1	Elastin-binding protein EbpS	Elastin-binding protein EbpS	plasma membrane; integral membrane	*ebpS* NWMN_1389	2	2	1	2	2
WP_000728056.1	Extracellular matrix protein-binding adhesin Emp	Extracellular matrix protein-binding protein Emp	cell surface	*ssp* NWMN_0758	2	2	−2	−3	−2
WP_000792564.1	Immunoglobulin-binding protein Sbi	Immunoglobulin-binding protein Sbi	extracellular region; plasma membrane	*sbi* NWMN_2317	2	1	−1	−2	−2
WP_000610306.1	MSCRAMM family adhesion SdrE	Serine-aspartate repeat-containing protein E	extracellular region; cell wall; integral membrane	*sdrE* NWMN_0525	−1	−2	−3	−1	−3
WP_000739209.1	Complement convertase inhibitor Ecb	Efb-c domain-containing protein	extracellular space	NWMN_1066	−3	−2	−1	−4	−3
WP_001549158.1	Extracellular adherence protein Eap/Map	65 kDa membrane protein (map-ND2C)	plasma membrane	NWMN_1872	−12	−13	−2	−10	−13
WP_001056178.1	MSCRAMM family adhesion clumping factor ClfA	Clumping factor A (Fibrinogen receptor A) (Fibrinogen-binding protein A)	extracellular region; cell wall; membrane	*clfA* NWMN_0756	−4	−3	−10	−8	−16

## Data Availability

All data generated or analysed during this study are included in this published article and its Supplementary Information Files. Furthermore, the mass spectrometry proteomics data have been deposited to the ProteomeXchange Consortium via the PRIDE [63] partner repository with Project accession: PXD025436.

## Supplementary Materials

The following supporting information can be downloaded at: https://www.mdpi.com/article/10.3390/microorganisms12081725/s1, Supplemental Information File S1—qPCR details. Supplemental Information File S2—Modified fractionation protocol. Supplemental Information File S3—Newman genomes. Supplemental Information File S4—Heatmap mutant-step. Supplemental Information File S5—Heatmap clinical-step. Supplemental Information File S6—Qualitative results—pilot step. Supplemental Information File S7—Quantitative results mutant step. Supplemental Information File S8—Quantitative results clinical step.
