# Supplementary material for: Surface-Shaving of Staphylococcus aureus Strains and Quantitative Proteomic Analysis Reveal Differences in Protein Abundance of the Surfaceome
Source: Microorganisms. 2024 Aug 21;12(8):1725. doi: 10.3390/microorganisms12081725 (PMC11357550; doi:10.3390/microorganisms12081725)
Supplement: Supplementary file 1 [file microorganisms-12-01725-s001.zip › Supplemental information File S4_Heatmap-mutants_240813.pdf]

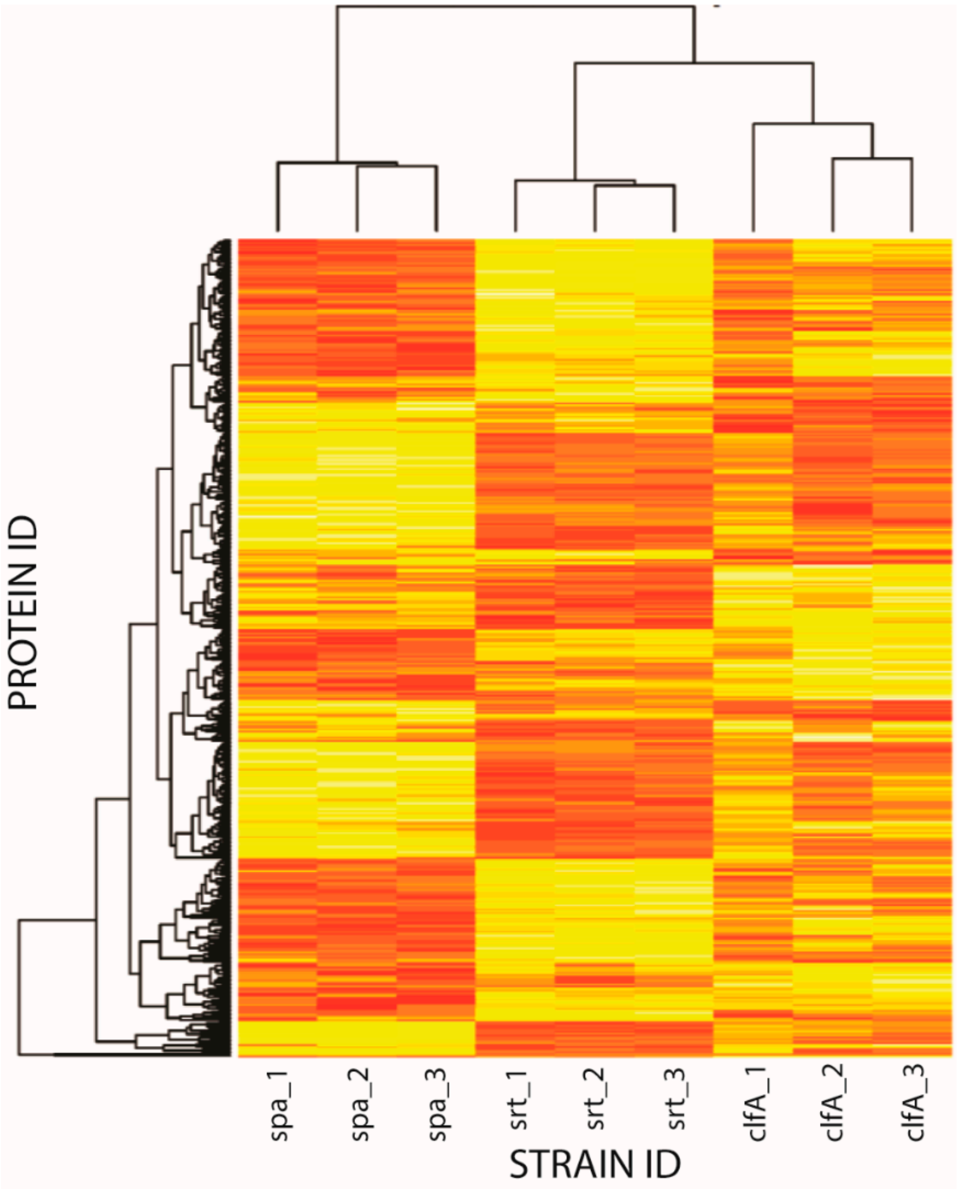

Supplemental information S4 Heatmap showing clustering of differential protein expression levels (fold changes) in the Δspa (spa_1, 2, 3), Δsortase (srt_1, 2, 3) and ΔclfA (clfA_1, 2, 3), compared to the Newman strain. The y-axis shows the protein IDs, whereas the x-axis shows the strain ID number for each individual strain analysis done in triplicates. Red colour indicates a higher expression level (fold change of strain compared to Newman), whereas white (weak yellow) signifies a lower expression level (fold change of strain compared to Newman). Clustering regarding similarity in high or low expression as compared to the Newman strain was allowed for the protein identification (PROTEIN ID), but was also allowed for the samples (STRAIN ID), strains showing similar patterns in the protein expression profiles cluster together and as can be seen the triplicate samples of the mutants cluster as they should. Here, in this cluster analysis, only values passing p-values< 0.05 from the ANOVA analysis is included.
