# Supplementary material for: Surface-Shaving of Staphylococcus aureus Strains and Quantitative Proteomic Analysis Reveal Differences in Protein Abundance of the Surfaceome
Source: Microorganisms. 2024 Aug 21;12(8):1725. doi: 10.3390/microorganisms12081725 (PMC11357550; doi:10.3390/microorganisms12081725)
Supplement: Supplementary file 1 [file microorganisms-12-01725-s001.zip › Supplemental information File S5_Heatmap-clinical-sorted_240813.pdf]

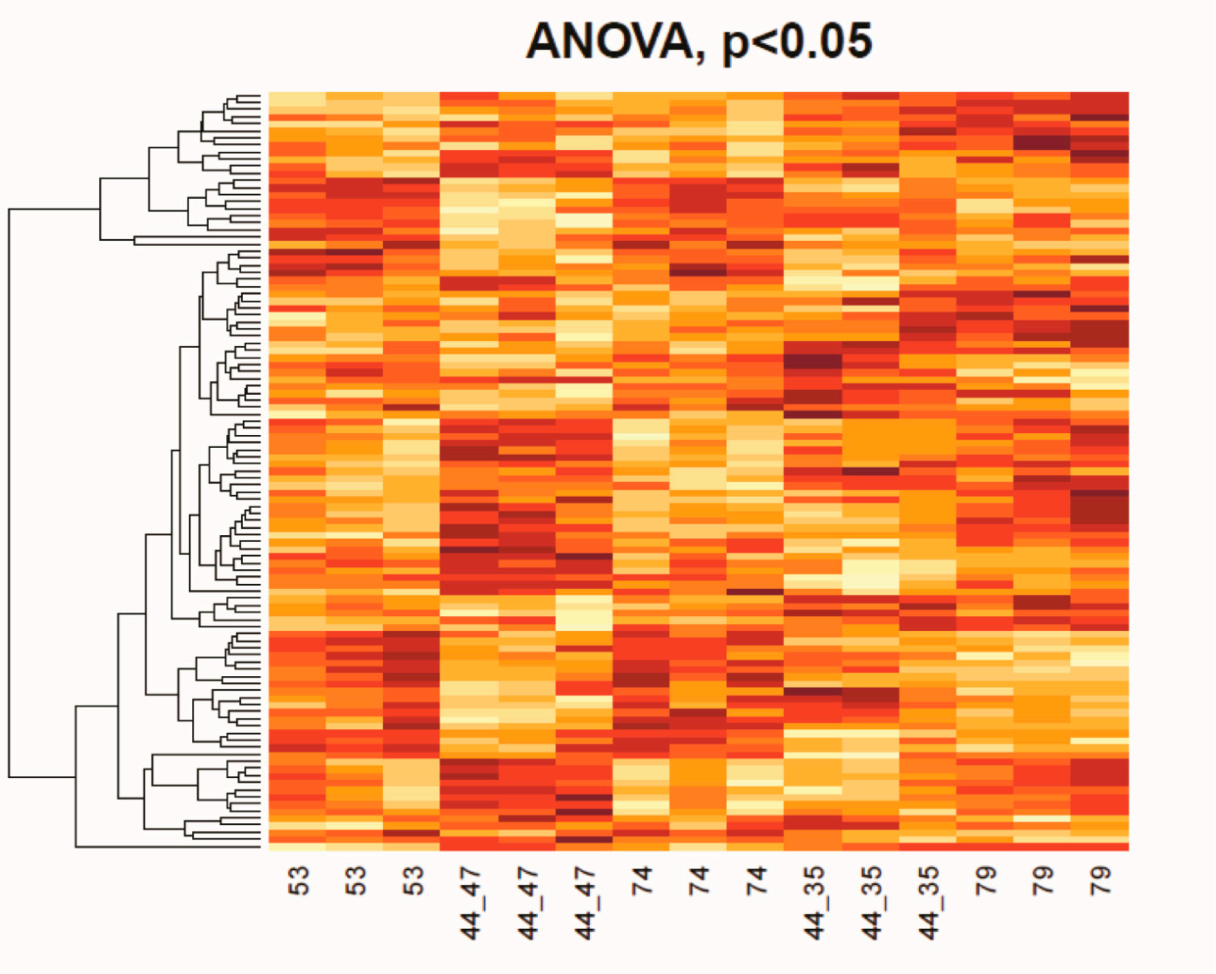

Supplemental information S5. Heatmap showing clustering of differential protein abundance (fold changes) for the Clinical strains study, showing the protein abundance of two clinical reference strains LS-1 (53) (1,2,3) and SH1000 (74) (1,2,3), and three clinical strains CCUG 74309 (44_35) (1,2,3), CCUG 74310 (44_47) (1,2,3) and CCUG 74311 (79) (1,2,3) when compared to the Newman strain. The y-axis shows the protein IDs, whereas the x-axis shows the strain ID number for each individual strain analysis done in triplicates. Red colour indicates a higher abundance, whereas yellow signifies a lower abundance (fold change (FC) of strain compared to Newman WT). Clustering regarding similarity in high or low abundance as compared to the Newman strain was allowed for the protein identification (PROTEIN ID) only. Here, in this cluster analysis, only values passing p-values < 0.05 from the ANOVA analysis is included.
