# Supplementary material for: Surface-Shaving of Staphylococcus aureus Strains and Quantitative Proteomic Analysis Reveal Differences in Protein Abundance of the Surfaceome
Source: Microorganisms. 2024 Aug 21;12(8):1725. doi: 10.3390/microorganisms12081725 (PMC11357550; doi:10.3390/microorganisms12081725)
Supplement: Supplementary file 1 [file microorganisms-12-01725-s001.zip › Supplemental_information File S2_Modified_fractionation_protocol_240813.pdf]

MODIFIED GRADIENT IN FRACTIONATION

Protocol according to manufacturer

High pH spin of TMT-labelled peptides Elution enough for 1 sample = 300 µl

FractionNo.	Acetonitrile(%)	Acetonitrile(µL)	Triethylamine(0.1%)(µL)
1	10.0%	100	900
2	12.5%	125	875
3	15.0%	150	850
4	17.5%	175	825
5	20.0%	200	800
6	22.5%	225	775
7	25.0%	250	750
8	50.0%	500	500

Modified protocol

TMT gradient enough for 3 samples = 1000 µl, wash 3%

FractionNo.	Acetonitrile (%)	Acetonitrile (µL)	Triethylamine (0.1%)(µL)
1	7.0%	70	930
2	9.0%	90	910
3	10.0%	100	900
4	11.0%	110	890
5	12.0%	120	880
6	14.0%	140	860
7	16.0%	160	840
8	18.0%	180	820
9	20.0%	200	800
10	22.0%	220	780
11	25.0%	250	750
12	50.0%	500	500
**13=extra**	75.0%	750	250
